# Limb trauma: the use of an advanced wound care device in the treatment of full-thickness wounds

**DOI:** 10.1007/s11751-013-0165-8

**Published:** 2013-07-30

**Authors:** L. Vaienti, A. Marchesi, G. Palitta, R. Gazzola, P. C. Parodi, F. Leone

**Affiliations:** 1 Department of Plastic Surgery, IRCCS Policlinico San Donato, San Donato Milanese, MI Italy; 2 O.U. of Plastic Surgery, Ospedale della Misericordia, Udine, UD Italy

**Keywords:** Upper limb, Lower limb, Hyaluronic acid, Full-thickness wound

## Abstract

This is an observational case series of 15 patients with full-thickness traumatic wound defects treated with a dermal substitute. There were 8 male and 7 female patients with a mean age of 36.6 years. Eight patients had trauma to the lower limbs and 7 were of the upper limbs, with the average lesion size 104.4 cm^2^ (range 6–490 cm^2^). The time to complete healing had a mean average time of 26.8 days (range 16–60 days). All patients went on to successful repair with 6 patients requiring a second application of the substitute and 5 patients needing split thickness skin grafts. Infection was recorded in one patient.

## Introduction

In the last few decades, there has been a search for solutions in tissue repair without need for autografts. This has produced a development of biological substitutes that could repair or improve the function of tissue. The use of these products enlarges the spectrum of therapeutic resources available for the treatment of skin lesions, thereby changing the approach to many diseases [1]. The advent of so-called advanced wound care dressings has revolutionized the treatment of soft tissue loss, especially in burns and difficult wounds, e.g., pressure and vascular diabetic ulcers, to become an essential resource.

Derivatives of hyaluronic acid (HA) are of particular importance within the field of dermal replacement products [2–7]. HA is the main degradation product in these substitutes and exerts many effects on wound healing, including maintenance of the homeostasis [8], enhancement of the angiogenesis [9] and organization of collagen deposition [10]. HA benefits epithelial regeneration also, and the HA macromolecule seems to have free-radical scavenging properties. HA derivatives are available in several esterified forms, one of which is HYAFF^®^ (benzyl esters of hyaluronic acid), a biopolymer organized in a three-dimensional scaffold that allows fibroblast colonization producing an ordered reconstruction of dermal tissue [11]. One of these recent HA derivatives is a dermal substitute composed by HYAFF, used in burn care practice as biological regenerative matrix on both deep partial-thickness and full-thickness burns and as temporary coverage of the wounds [1, 12]. We have looked at the efficacy of this substitute, originally developed for dermal regeneration in different full-thickness wounds, for cases of limb trauma with exposed bone or tendon which continue to be challenging problems for plastic surgeons. We have undertaken this observational study to evaluate the clinical efficacy and safety of the product on dermal regeneration in cases of severe soft tissue loss.

## Materials and methods

This prospective case series included 15 patients with full-thickness wounds caused by trauma which were treated with the dermal substitute; the series was accrued from June 2007 to May 2010. All patients were informed about product use and consented to the study. There were 8 male and 7 female patients with a mean age of 36.6 years. Eight patients had trauma to the lower limbs and 7 were of the upper limbs, with the average lesion size 104.4 cm^2^ (range 6–490 cm^2^), (Table 1). Twelve patients had exposed tendons and two had exposed bone (no area of bone exposure exceeded 2 cm^2^). The causes of trauma were, in ten cases (66 %), accidents at work or home; in three cases (20 %), burns and in two cases (13.3 %), surgical wounds. All cases were treated urgently, and none of the patients was affected by comorbidities. Evaluation of inflammatory indexes (white cells, CRP and ESR) were carried out at 3, 6, 10 days and 4 weeks after the application of the product. The assessment of clinical epithelialization and the aesthetic quality of the newly formed skin (skin texture, color similarity and elasticity) were performed at least 6 weeks after product application. In six patients (40 %), a second application was necessary. The layer was left on the wounds for a mean time of 15.9 days (range 14–21). Any adverse events and complications were recorded.

**Table 1:** Demography and baseline characteristics

N°	Age (years)	Sex	Trauma localization	Cause	Lesion area^a^ (cm^2^)	Bone exposure	Tendon lesion	Hyalomatrix^®^ PA (*n*)	Hyalomatrix^®^ PA residence time (days)
1	45	F	Leg, third distal	Accident	20 × 8 (160)	+(2 × 1 cm)	+	2	21 + 21
2	33	M	Hand	Work accident	16 × 6 (96)	–	+	1	14
3	17	M	Hand	Surgery	3 × 6 (18)	–	+	1	14
4	19	M	Forearm	Work accident	4 × 8 (32)	–	+	1	14
5	62	M	Leg, third distal	Accident	13 × 9 (117)	–	+	2	21 + 21
6	42	F	Hand	Home accident	6 × 4 (24)	–	+	1	14
7	34	F	Foot	Accident	3 × 4 (12)	–	+	1	14
8	29	F	Foot	Accident	6 × 7 (42)	–	+	1	14
9	3	M	Hand	Burn	5 × 5 (25)	–	–	2	14 + 14
10	48	M	Foot	Work accident	12 × 4 (48)	–	+	2	14 + 14
11	47	F	Hand	Burn	3 × 2 (6)	–	–	1	14
12	51	M	Leg, third distal	Surgery	35 × 14 (490)	+(2 × 1 cm)	+	2	21 + 21
13	68	F	Thigh	Burn	20 × 16 (320)	–	–	2	14 + 14
14	22	F	Leg, third distal	Accident	9 × 4 (36)	–	+	1	14
15	30	M	Forearm	Work accident	20 × 7 (140)	–	+	1	14

^a^Lesion area by maximum length and width

### Product application procedure

Hyalomatrix^®^ PA is designed as a sterile, bi-layered wound dressing. It is a biodegradable, absorbent, non-woven pad entirely composed of HYAFF^®^ which constitutes the wound contact layer. The HYAFF^®^ layer is physically coupled with a transparent and flexible film of a medical-grade synthetic elastomer that acts as a semipermeable barrier against external contaminants. As the product is applied on the wound bed, the HYAFF^®^ wound contact layer provides a three-dimensional scaffold that permits the colonization of the product by fibroblasts and onto which extracellular matrix components are laid down, resulting in an almost ordered reconstruction of the dermal tissue. The transparent elastomeric film, characterized by a vapor transmission rate comparable to that of normal skin, prevents excessive body fluid loss. The product conforms well to wound edges and can be cut to suit the shape of the lesion.

All wounds were subjected to a presurgical debridement and assessment of the lesion. Hemostasis of the visible bleeding points was carried out. Once a clean wound bed was obtained, an adequate-sized pad was shaped to the wound and applied with the HYAFF^®^ scaffold in direct contact with the wound bed and contoured to the edges, sutured with nylon sutures and dressed with sterile cotton gauze. An elastic bandage was applied for a non-compressing cover. After a period of not less than 15 days, a complete inspection and assessment of the wound was performed by removing the silicon film. Depending on the achievement of the desired level of granulation tissue, it was decided if a new application of the product was necessary. If a wound bed with a good blood supply was observed, repeated washing with saline was carried out and dressing with paraffin gauze followed by cotton gauze and a bandage applied. Patients returned for a change of dressings weekly. Swabs were taken for bacteriologic culture before application and thereafter at weekly intervals during wound inspection. Antibiotic prophylaxis (Cefazolin) was administered from the day of the product application for 3 days until microbiological results were available. Each patient with clinical evidence of infection received appropriate antibiotic therapy (based on results of cultures), and dressing changes were increased to twice per week until the infection settled. Weekly dressing changes continued until epithelial healing was complete. Regular photographic documentation was taken before, during and after treatment.

## Results

**Fig. 1:**
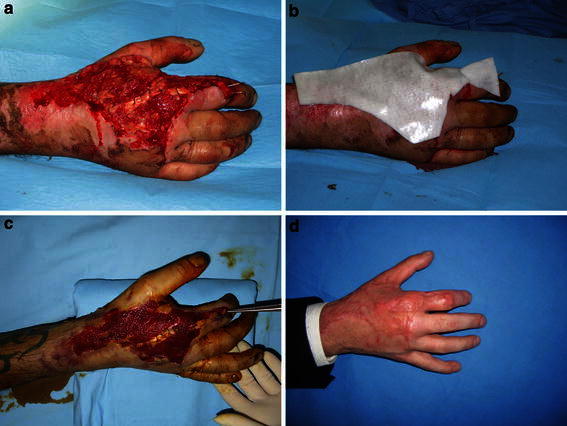
Patient with traumatic injury of the right hand. **a** Lesion after surgical debridement, **b** application of Hyalomatrix^®^ PA, **c** lesion after the Hyalomatrix^®^ removal, **d** lesion completed healed (after 30 days)

**Fig. 2:**
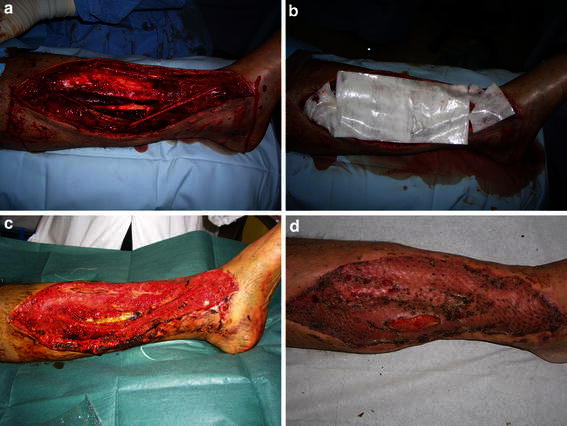
Patient with a lower limb traumatic lesion. **a** Lesion after surgical debridement, **b** application of Hyalomatrix^®^ PA, **c** lesion after Hyalomatrix^®^ PA removal: the newly formed tissue is evident, **d** after one month: >90 % taking of the meshed-skin graft

All treated cases (*n* = 15) had satisfactory tissue repair (Table 2). Out of 15 cases, 6 (40 %) needed a second application of the product in order to obtain the desired wound bed. Of the six, 3 had lesion sizes ranging from 117 to 390 cm^2^ needing autologous skin grafts to complete wound cover. Ten lesions (67 %) closed spontaneously by re-epithelialization (Fig. 1), but 5 cases (33 %) needed autologous skin grafts to complete the epithelialization process (Fig. 2). The time to complete healing had a mean average time of 26.8 days (range 16–60 days). The inflammatory indexes were normal in 13 cases (87 %) while in two cases (13 %) were positive. The assessment of the quality of the new skin was optimal for 5 cases (33.3 %), good in 7 (46.6 %) and modest in 3 (20 %). In one case (6.7 %), the onset of infection was observed, and this event delayed the epithelialization process; in this case, the second application of the product was postponed until a decrease in the inflammatory markers. No other complications were noticed during the product application and duration of the study.

**Table 2:** Efficacy and safety results

N°	Inflammatory indexes^a^	Skin autograft	Time to complete healing (days)	Quality of the skin^b^	Complications
1	Negative	Yes	28	Moderate	–
2	Positive	No	30	Good	–
3	Positive	No	21	Optimal	–
4	Positive	Yes	21	Good	–
5	Positive	Yes	28	Optimal	–
6	Positive	No	23	Good	–
7	Positive	No	21	Optimal	–
8	Positive	No	16	Optimal	–
9	Positive	No	30	Normal	–
10	Positive	No	28	Good	–
11	Positive	No	16	Moderate	–
12	Negative	Yes	60	Moderate	Infection
13	Positive	Yes	28	Good	–
14	Positive	No	30	Good	–
15	Positive	No	23	Good	–

^a^Evaluation of inflammatory indexes (white cells, C-RP and ESR) were carried out at 3, 6, 10 days and 4 weeks after the application of Hyalomatrix^®^ PA

^b^Evaluation at least 6 weeks after Hyalomatrix^®^ PA application (skin texture, skin color similarity and elasticity)

## Discussion

Limb injuries creating exposed bone or tendon continue to be challenging problems for plastic surgeons. There is now a better understanding of vascular anatomy of the lower leg and better use of improved wound care technology which has led to judicial use of flaps and delayed closures. Severe limb trauma has significant socio-economic effects; there is considerable expenditure in terms of direct costs (orthopedic implants and devices, dressing materials, antibiotics) and indirect costs (lost working days, family involvement, transportation, etc.). For all these reasons, we have decided to evaluate treatment of traumatic lesions of the limbs with an advanced wound care dressing which proposes to promote and sustain effective coverage of bony and tendon structures and, simultaneously, to accelerate the healing process through an induction of the dermal component.

A previous clinical study with a product based on Hyaluronic acid was carried out on deep partial and full-thickness burns in young and adult patients [13]. In contrast, we have utilized this dermal substitute in severe extremity injuries, although less life-threatening than burns but still present as a difficult soft tissue problem for limb salvage. These results suggest the product does induce the production of new granulation tissue suitable for receiving a new epithelial cover.

The effective protection provided by the layer of silicon from infections was supported by the incidence of 1 case with infection (6.7 %) in this series. The outer silicone membrane barrier may limit colonization and therefore protect the hyaluronan from protease digestion.

During this study, despite the transparent nature of the external silicon layer, we were not able to verify specific changes on the wound bed until removal of the product around the fourteenth day of treatment. This observational study has confirmed some remarkable induction capabilities of this dermal substitute on producing granulation tissue suitable for split skin grafts. Although pain relief was demonstrated for the intra-articular introduction of hyaluronic acid derivatives in the treatment of osteoarthritis [14, 15], we have not measured this clinical effect in the patients in this study. Myers et al. [16–19] have proposed a role of HA in the induction of angiogenesis in granulation tissue. In a previous clinical study, Price et al. [20, 21] have suggested that Hyalomatrix^®^ might function as a dermal regenerative template because of its high level of esterification in HYAFF^®^, and this is supported by the observation of early wound epithelialization. This study has shown satisfactory results with this approach, but it stressed the importance that close monitoring of wound progress is made. For these reasons, we believe that a hyaluronic acid-derived dermal substitute can be indicated for non-invasive treatment of complicated traumatic wounds where there is need for a dermal inductive effect to cover important structures and a need of protection from infections [13]. The quality of the neo-dermal tissue is clinically better than dystrophic scar tissue that would be obtained from healing by secondary intention, but it is not yet comparable to that of the original healthy skin. On certain locations such as hands, knees, elbows and ankles, use of this substitute will need longer-term follow-up for the possible onset of contractures which could affect function. Finally, with this approach to wound cover, there is need for high patient compliance and specific physiotherapy aimed at functional recovery after the immobilization of the treated area.

## Conclusion

This study has evaluated the performance of a dermal substitute for the cover of wounds to limbs after trauma. Successful cover was achieved in all cases, with the need of additional split thickness skin grafts in 33 % of patients. Infection was encountered in one patient.
